# The elusive ligand complexes of the DWARF14 strigolactone receptor

**DOI:** 10.1093/jxb/ery036

**Published:** 2018-01-31

**Authors:** Gunilla H Carlsson, Dirk Hasse, Francesca Cardinale, Cristina Prandi, Inger Andersson

**Affiliations:** 1 Laboratory of Molecular Biophysics, Department of Cell and Molecular Biology, Uppsala University, Husargatan, Uppsala, Sweden; 2 Department of Agricultural, Forestry and Food Science, University of Turin, Largo Paolo Braccini, Grugliasco, Italy; 3 Department of Chemistry, University of Turin, via P. Giuria, Torino, Italy

**Keywords:** Crystal structure, DWARF14, ligand binding, *Oryza sativa*, strigolactone, strigolactone receptor

## Abstract

Strigolactones, a group of terpenoid lactones, control many aspects of plant growth and development, but the active forms of these plant hormones and their mode of action at the molecular level are still unknown. The strigolactone protein receptor is unusual because it has been shown to cleave the hormone and supposedly forms a covalent bond with the cleaved hormone fragment. This interaction is suggested to induce a conformational change in the receptor that primes it for subsequent interaction with partners in the signalling pathway. Substantial efforts have been invested into describing the interaction of synthetic strigolactone analogues with the receptor, resulting in a number of crystal structures. This investigation combines a re-evaluation of models in the Protein Data Bank with a search for new conditions that may permit the capture of a receptor–ligand complex. While weak difference density is frequently observed in the binding cavity, possibly due to a low-occupancy compound, the models often contain features not supported by the X-ray data. Thus, at this stage, we do not believe that any detailed deductions about the nature, conformation, or binding mode of the ligand can be made with any confidence.

## Teaser

A critical survey of strigolactone receptor–ligand structures available in the literature shows that the models frequently contain features not supported by the X-ray data.

## Introduction

Strigolactones (SLs) are phytohormones that control many aspects of plant growth. SLs are produced mainly in the roots, which they shape morphologically, especially in relation to nutrient deprivation. They are also transported to and, to a limited extent, synthesized in the shoots, where they exert pleiotropic functions in development and stress responses, most prominently repression of axillary bud outgrowth (Gomez-Roldan *et al.*, 2008, Umehara *et al.*, 2008). As exogenous signals, SLs promote the symbiotic association between land plants and arbuscular mycorrhizal fungi that improve nutrient uptake (Akiyama *et al.*, 2005; Bouwmeester *et al.*, 2007; Al-Babili and Bouwmeester, 2015). Secreted into the soil, SLs can also function as germination signals for parasitic plants and may cause considerable crop failure. For instance, parasitic weeds such as witchweed (*Striga* sp.) and broomrape (*Orobanche* sp.) have become completely dependent on this interaction for germination of their seeds (Cook *et al.*, 1966; Cardoso *et al.*, 2011).

Despite their relatively recent discovery as plant hormones (Gomez-Roldan *et al.*, 2008; Umehara *et al.*, 2008), rapid progress has been made in understanding SL function, including the identification of a signalling pathway for SLs. This pathway involves the SL receptors of the DWARF14 (D14) class of α/β-hydrolase enzymes, the MORE AXILLARY GROWTH2 (MAX2) F-box proteins, and the SUPPRESSOR OF MAX2 1 (SMAX1) and SMAX1-LIKE (SMXL) family of chaperonin-like proteins. In *Oryza sativa* (rice), MAX2 proteins interact with D14 in an SL-dependent manner (Zhou *et al.*, 2013) and act as part of a Skp, Cullin, F-box-containing complex (or SCF complex) that is assumed to regulate ubiquitination and degradation of target proteins. The SMXL proteins are targets of MAX2/D14-dependent protein degradation. This pathway appears to be evolutionarily conserved; homologues of these proteins have also been identified in, among others, Arabidopsis (*Arabidopsis thaliana*), pea (*Pisum sativum*), and petunia (*Petunia hybrida*) (Arite *et al*, 2009; Waters *et al.*, 2012; Hamiaux *et al.*, 2012; reviewed in Bennett and Leyser, 2014).

There are notable similarities between SLs and their receptors and the perception and signalling mechanisms of several other plant hormones, including auxins (Hayward *et al.*, 2009) and gibberellins. The main analogies include response triggering by proteasome-mediated destruction of ubiquitinated repressors, and the central role of F-box protein adaptors in this process. Similarities extend to the involvement of members in the α/β-hydrolase protein family as receptors for hormonal ligands, such as in the case of the Gibberellin Insensitive Dwarf1 (GID1) protein for bioactive gibberellins (Sun, 2010). However, unlike the gibberellin receptor GID1, which is enzymatically inactive and stably interacts with its target hormone, SL receptors destroy their target.

In rice, the SL signal is transmitted through interaction with a D14 receptor, the DWARF3 (D3, orthologue of MAX2 in Arabidopsis) F-box protein and the DWARF53 (D53) protein, which has been identified as a target protein (Jiang *et al.*, 2013; Zhou *et al.*, 2013). The D14 α/β-hydrolase enzyme uses a Ser–His–Asp catalytic triad to perceive and hydrolyse SLs simultaneously. Recent results (de Saint Germain *et al.*, 2016; Yao *et al.*, 2016) indicate that a covalent bond with histidine may form upon hydrolysis of the active hormone at the receptor.

A major challenge is to identify the active form of SLs and their molecular mode of action. On interaction with D14, the synthetic SL analogue GR24 is hydrolysed to two compounds (Hamiaux *et al.*, 2012; Nakamura *et al.*, 2013), a tricyclic lactone (the ABC ring moiety), and 5-hydroxy-3-methylbutenolide (the D-OH ring), or possibly some other degradation intermediate such as 2,4,4-trihydroxy-3-methyl-3-butenal (TMB; Zhao *et al*, 2013). This hydrolysis is relatively slow (Hamiaux *et al.*, 2012; de Saint Germain *et al.*, 2016), raising hopes that a complex between the receptor and the hydrolysed product may be captured in a crystal structure. However, the challenges involved in such studies are numerous and include issues such as the low contribution to the total scattering of the ligand compared with the protein partner, the high concentration of components used for the purification, crystallization, and cryo-cooling that may often outcompete the desired ligand, and importantly the incompatibility of ligand binding with the packing of the protein in the crystal lattice. These questions will be elaborated further below.

We searched for conditions that may allow the crystallization of a complex between rice D14 and SL analogues, such as GR24, and have compared the resulting structures with the structures of putative complexes deposited in the databases. The results from our survey may serve as an illustration of critical issues in protein–ligand crystallization efforts for the D14–SL complex.

## Materials and methods

### DNA cloning

The *Os*D14 cDNA fragment lacking the sequence encoding the first 51 N-terminal amino acids was amplified from a cDNA pool obtained from roots of *Oryza sativa* (*Japonica* subgroup) by PCR using the Phusion^®^ High-Fidelity PCR Master Mix (Thermo Fisher Scientific) with forward primer 5'-GGATCCAGCGGGGCGAAGCTGCT-3' (*Bam*HI site underlined) and reverse primer 5'-GGTACCTTAGT ACCGGGCGAGAG-3' (*Kpn*I site underlined). The PCR product was analysed by agarose gel electrophoresis. The eluted D14 PCR fragment was prepared for TA cloning by adding 3'-adenine ends using PCR Master Mix (Thermo Fisher Scientific) and cloned into pGEM-T (Promega). After confirming the correct D14-short coding sequence by Sanger sequencing, the fragment was cloned into the protein expression plasmid pHUE (Catanzariti *et al.*, 2004) by using the endonuclease restriction sites *Bam*HI and *Kpn*I. The resulting construct (*Os*D14-short) consists of *Os*D14 lacking the first 51 amino acids, fused at the N-terminus to ubiquitin carrying an N-terminal His-tag and a C-terminal cleavage site for a ubiquitin-specific peptidase 2 (Usp-2). The reactive serine in the active site of *Os*D14 was substituted by an alanine (S147A). Mutation was achieved by using the Quick Change II Site-Directed Mutagenis Kit (Agilent Technologies) and reactions were performed as described in the user’s manual with oligos 5'-CAC TCC GTC GCC GCC ATG ATC GGC ATC-3' (sense) and 5'-GAT GCC GAT CAT GGC GGC GAC GGA GTG-3' (antisense). The expression vector pHUE-*Os*D14-short was used as template for the PCR. Successful introduction of the mutation was confirmed by DNA sequencing.

### Protein expression

*Os*D14 (short, tagged version as described above) was heterologously expressed in *Escherichia coli* strain BL21 DE3 (Life Technologies). After transformation with *Os*D14-short, *E. coli* was grown at 37 °C in 2× YT medium (1.6% tryptone, 1% yeast extract, 0.5% NaCl) containing 1 mM betaine and 100 μg ml^−1^ ampicillin until OD_600_=0.6 was reached. Cells were then cooled to 20 °C, induced with 0.1 mM isopropyl-β-d-thiogalactopyranoside (IPTG), and cultivated overnight at 20 °C.

### Protein purification

The cells were harvested by centrifugation and re-suspended in 10% glycerol, 20 mM Bis-Tris propane pH 8, 0.5 M NaCl (Buffer A). After incubation with DNase, the cells were lysed using a cell disruptor CF Range (Constant Systems Ltd) at 35 kpsi.

The cell lysate was applied to a gravity flow Ni-NTA column (Bio-Rad), washed with 50 mM imidazole in Buffer A, and eluted with 500 mM imidazole in Buffer A. After lowering the salt concentration by diluting the eluate with 20 mM Bis-Tris propane pH 8 followed by a protein concentrating step using a Vivaspin centrifugal concentrator (Sartorius), the protein was incubated at 37 °C with 0.1 mg ml^–1^ Usp-2 (Catanzariti *et al.*, 2004) to remove the His-ubiquitin tag. In the subsequent step, the His-ubiquitin tag and the His-tagged Usp-2 protease were removed from *Os*D14 by applying the solution a second time to an Ni-NTA column. Prior to this step, imidazole was removed using a PD10 column (GE Healthcare) equilibrated with Buffer A. The eluate containing the cleaved *Os*D14 was collected, diluted 30 times in buffer MQ (5% glycerol, 20 mM Bis-Tris propane pH 9.0), and applied to a 1 ml MonoQ column equilibrated with MQ buffer. *Os*D14 was eluted with a gradient of 0–0.5 M NaCl in MQ buffer. Fractions containing *Os*D14 eluted at ~0.1 M NaCl were pooled and concentrated to 2–4 mg ml^−1^. The mutant S147A was purified in the same manner as the native *Os*D14.

### Crystallization and data collection

*Os*D14 (lacking the N-terminal 51 amino acids) was crystallized at room temperature (293 K). Crystallization conditions were screened using commercial screens and a mosquito^®^ Crystal robot (TTP Labtech) with 0.2 μl drop size. Initial crystals were obtained in 10% 2-methylpentanediol (MPD), 0.1 M HEPES pH 7.0 (pHClear Suite, Qiagen). These crystals belong to space group *P*2_1_2_1_2_1_ with unit cell parameters *a*=48.1, *b*=88.6, *c*=118.9 Å, α, β, γ=90°, two molecules in the asymmetric unit, and 42% solvent content. Data were collected to 1.35 Å resolution at the ESRF beam line ID23-2. In the following, to exclude potential ligands (MPD and HEPES), extensive screening was made with alternative buffers and precipitating agents at 293 K using the hanging drop vapour diffusion method. Crystals of apo *Os*D14 were obtained using polyethylene glycol (PEG) 6000 at concentrations of 5–30% and in the pH range 6.0–9.5. These conditions were subsequently tried for crystallization of complexes of native *Os*D14 and a S147A mutant with SL analogues by soaking and co-crystallization. We used a number of compounds of various sizes (Prandi *et al.*, 2011; Artuso *et al.*, 2015, Supplementary Fig. S1 at *JXB* online), including GR24 [a racemic mixture of (+)-GR24 and (–)-GR24]. The compounds were dissolved in acetone to a concentration of 0.2 M, and either added to a drop containing a crystal for soaking experiments or to the solution of *Os*D14 prior to crystallization for co-crystallization experiments. Incubation times were 20–60 min. Prior to data collection, crystals were soaked in a reservoir solution containing cryo-protectant (30% glycerol and any analogue present in the crystallization droplet), transferred into a nylon loop (Hampton Research), flash-cooled in liquid nitrogen, and maintained at 100 K for data collection. Data collection statistics are presented in Table 1.

**Table 1.** Data collection and refinement statistics

	*Os*D14 MPD complex
Protein Data Bank id	6ELX
Data collection	
Beam line	ESRF ID23-2
Wavelength (Å)	0.9919
Space group	*P*2_1_2_1_2_1_
Wavelength (Å)	0.9919
Unit cell parameters (Å)	*a*=48.1 *b*=88.6 *c*=118.9; α, β, γ,=90°
Solvent content (%)	42.0
Resolution (Å)	44.61–1.35 (1.40–1.35)
No. of observations	516 416 (36044)
No. of unique reflections	107 535 (8569)
Completeness (%)	95.72 (77.32)
Multiplicity	4.8 (4.2)
*R*_merge_	0.0510 (0.4184)
*R*_meas_^*a*^	0.0572 (0.4757)
<I/(σI)>	16.15 (2.91)
CC1/2	0.999 (0.813)
Refinement	
Resolution range (Å)	44.61–1.35 (1.40–1.35)
No. of reflections	107 538 (8569)
*R*_work_^*b*^	0.159 (0.217)
*R*_free_^*c*^	0.177 (0.229)
No. of atoms	
Protein	4388
Ligands	58
Waters	607
Average *B*-values (Å^2^)	16.58
Estimated from Wilson plot	11.96
Protein	14.76
Ligands	32.73
Solvent	27.74
Rms deviations from ideal values	
Bond lengths (Å)	0.006
Bond angles (°)	1.19
Ramachandran analysis^*d*^	
Favoured (%)	98.1
Allowed (%)	1.9
Outliers (%)	0.0

Values in parentheses are for the outer resolution shell.

^*a*^ *R*_meas_=∑_*h*_ ∑_*l*_ (n_*h*_/n_*h*_–1)^1/2^ | *I*_*hl*_–<*I*_*h*_> |/∑_*h*_ ∑_*l*_ <*I*_*h*_> (Evans, 2006; Evans and Murshudov, 2013).

^*b*^ *R*_work_=∑_*hkl*_| |*F*_o_|–|*F*_c_| |/∑_*hkl*_ |*F*_o_| where *F*_o_ and *F*_c_ are the observed and calculated structure factor amplitudes, respectively.

^*c*^ *R*_free_ calculated from a randomly chosen 5% of all unique reflections.

^*d*^ From ‘MolProbity’ (Chen *et al.*, 2010).

### Structure determination and refinement

The X-ray diffraction data were processed, scaled, and merged with the XDS suite (Kabsch, 2010) with 5% of the data set aside for *R*_free_ calculations. Phases were obtained by molecular replacement in Phenix (Adams *et al.* 2010) using the structure of RbsQ (PDB id 1wom; Kaneko *et al.*, 2005) as a search model. Refinement of the initial model was performed with Phenix alternated with inspection and manual rebuilding in Coot (Emsley *et al.*, 2010). Solvent molecules were identified using the water insertion command in Phenix and assigned based on peak heights of residual electron density, hydrogen-bonding patterns, and *B*-factors. Refinement statistics are presented in Table 1. The initial model was then used as a search model to solve all subsequent structures.

### Analysis of deposited structures

A structural superposition of *Os*D14 with deposited structures in the PDB was performed with the least-squares superposition function in O (Jones *et al.*, 1991). The default distance cut-off limit of 3.8 Å was used.

Initial σ_A_-derived maximum-likelihood electron density maps, 2*mF*_o_–*DF*_c_ and *mF*_***o***_–*DF*_c_ (Read, 1986, Pannu and Read, 1996), where *m* is the figure of merit and *D* is the Luzzati factor (Luzzati, 1953), were calculated using the deposited models and diffraction data downloaded from the PDB. The 2*mF*_o_*–DF*_c_ maps were coloured blue (or white) and contoured at 1.0 sigma (i.e. 1.0 SD above the mean electron density) and show where the model is expected to be, whereas the *mF*_o_*–DF*_c_ maps were coloured green (3.0 sigma) and red (–3.0 sigma); the green mesh shows where atoms are missing in the current model, while the red mesh shows where atoms are present in the model but not in the crystal. The initial maps were calculated without any prior manipulation or refinement of the deposited co-ordinates. In a second step, omit maps were calculated in the same manner after excluding the ligand from the calculation. Thirdly, after inspection of native and omit maps, in the relevant cases a suitable ligand was built into the positive difference density appearing at the 3.0 sigma level followed by 10 cycles of maximum likelihood restrained refinement in REFMAC5 (Murshudov *et al.*, 2011) or Phenix (Adams *et al.*, 2010). During this operation, care was taken to use the programs and scripts of the originators, as described in the respective PDB header.

The figures were created with ‘PyMOL’ (Version 1.6.0.0, Schrödinger, LLC).

## Results and Discussion

### Structure solution and overall structure

**Fig. 1.**
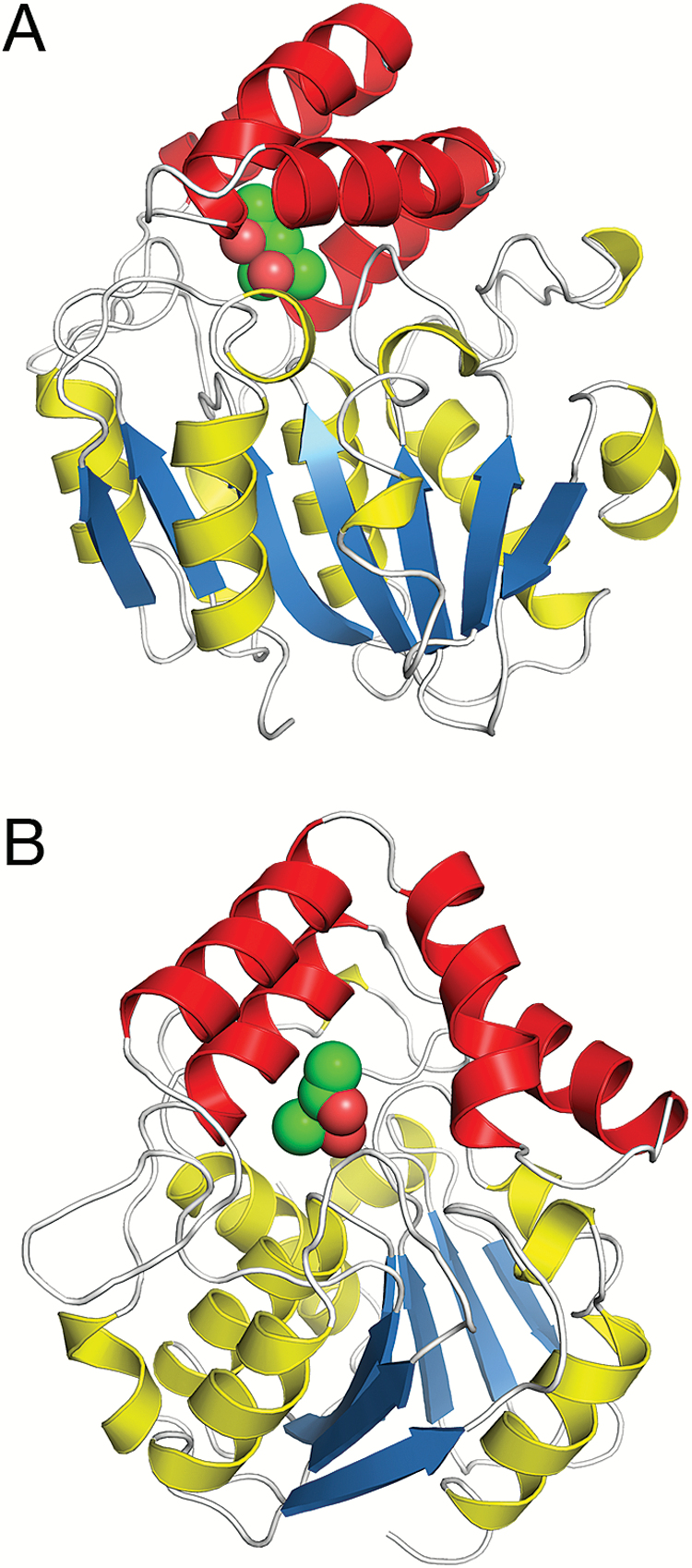
(A) Overall structure of *Os*D14. β-Strands of the core are coloured blue, associated helices yellow, and helices in the helical cap red. MPD is depicted as green spheres. (B) Binding of MPD in the ligand-binding cavity showing the opening of the cavity towards the solution. The two views in (A) and (B) are related by a 90° rotation with respect to the vertical axis.

The structure of *O. sativa* Dwarf14 (*Os*D14) was determined by X-ray crystallography to a resolution of 1.35 Å from a construct lacking the first 51 residues. The N-terminal 51 residues comprise an unusual combination of repeated glycine and serine residues, suggesting that this part may be flexible in the mature protein and/or that it may be glycosylated. *Os*D14 has a α/β-hydrolase fold (Gaiji *et al.*, 2012) with a seven-stranded β-sheet core domain flanked by seven α-helices, of which two are shorter 3_10_-helices. The short version of the rice D14, lacking the first 51 residues, starts with a helix, which is also present in the α/β-hydrolase structure RbsQ (PDB id 1wom) that was used as a search molecule for the structure solution. The central seven-stranded sheet of *Os*D14 is built of six parallel strands with the first N-terminal strand running antiparallel to the core (Fig. 1A). Two of the flanking helices are located on one side of the sheet and the remaining five reside on the other side. Inserted between strands five and six of the central sheet are four helices connected to the core by a β-hairpin loop on its N-terminal side and a shorter loop on the other side. This domain, commonly named the helical cap, forms a V-shaped lid over a deep cavity, which is assumed to bind the SL hormone (Fig. 1B). The cavity is relatively large and lined by hydrophobic residues. At the bottom of the cavity is a canonical catalytic triad consisting of Ser147, His297, and Asp268.

### Binding cavity

**Fig. 2.**
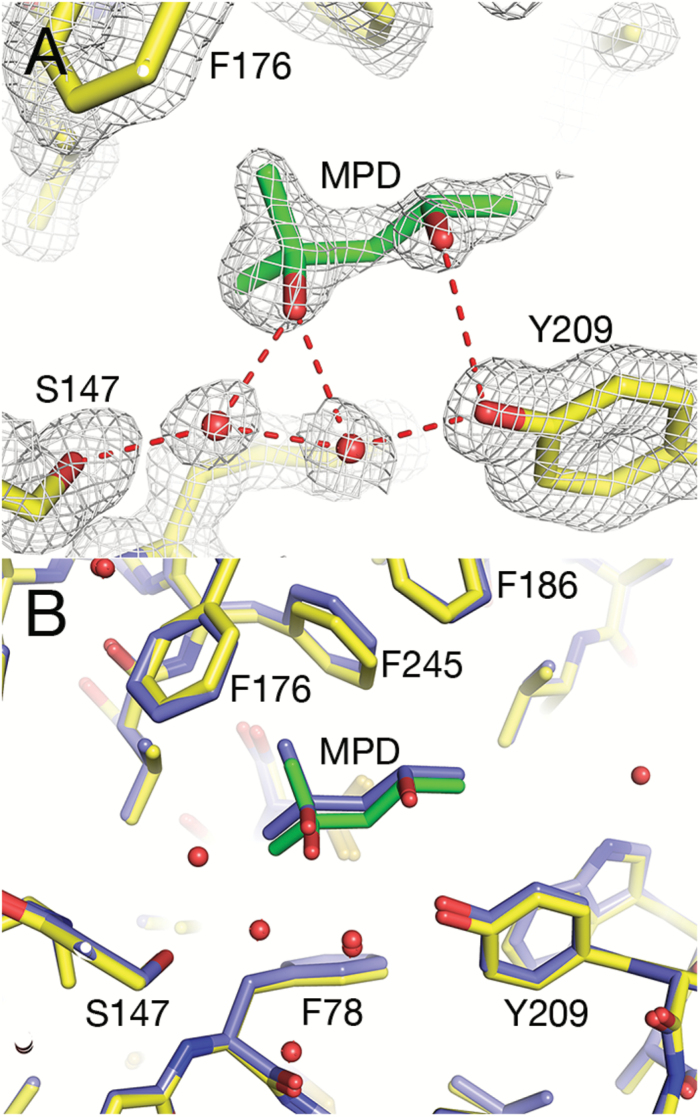
Binding cavity of *Os*D14. (A) Electron density around the ligand-binding site of *Os*D14, showing MPD built into the density. 2*mF*_o_–*DF*_c_ map contoured at 1.0 sigma (white) (B) Superposition of *Os*D14 with the structure of *O. sativa* D14 in PDB id 3wo4 (coloured blue).

After model building of the protein, residual density was observed in the ligand-binding cavity. We identified this as originating from MPD, which was used as a precipitating agent during crystallization. MPD was added as a racemic mixture of (4*S*)-2-methyl-2,4-pentanediol and (4*R*)-2-methyl-2,4-pentanediol, and it appears that both stereoisomers were able to bind in the cavity. Judging from the electron density, there was a preference for binding the 4*S* stereoisomer in one subunit (arbitrarily named molecule B in the PDB co-ordinate file). MPD is located some distance away from the catalytic triad, forms several hydrophobic interactions with residues lining the cavity (Phe78, Phe176, Phe186, Val194, Val244, and Phe245), and hydrogen-bonds to the hydroxyl oxygen of Tyr209 and to Oγ of Ser147 and Nε2 of His297 via a water molecule (Fig. 2A). In the second subunit (named molecule A), the binding of the 4*R* stereoisomer appears to be preferred, and the direct interaction with the Tyr209 hydroxyl group is replaced by a solvent-mediated contact. However, most probably there was a (unequal) mixture of the two stereoisomers binding at both sites. At this data resolution, estimating the contribution of the individual stereoisomers to each site is not feasible. The helical cap covers MPD like a dome, but is open to solvent on one side in this conformational state of the receptor (Fig. 1B). The undesired binding of MPD in the ligand-binding cavity of *Os*D14 illustrates a common problem in crystallographic investigations of ligand binding to proteins, as elaborated below.

### Difficulties in the crystallographic validation of structures of protein–ligand complexes

The interpretation of structures from electron density maps involves some level of subjectivity (Brändén and Jones, 1990), the extent of which depends on, for example, the quality of the X-ray data, the resolution, inherent disorder, and the experience of the interpreter. The now common practice of depositing the co-ordinates and the structure factor amplitudes in the PDB, which are then subjected to curation and analysis, has helped to raise the awareness of such errors. To aid this process, there are a number of programs available for validation and analysis of macromolecular structures (Read, 2011), which have helped to reduce significantly the number of erroneous protein structure models.

Validation of structures of protein–ligand complexes remains more problematic because of the infinitely variable chemical character of ligands that bind to protein, such as cofactors, substrate analogues, products, inhibitors, crystallization additives, etc., compared with the limited repertoire of the amino acids that constitute the protein structure. Common indicators of quality such as geometry and *R* factors work less well for small ligands; the geometry of small ligands is often poorly determined, and the low contribution of the ligand to the total scattering compared with that of the protein partner makes *R* factors a blunt indicator in this case. In addition, ligand occupancy depends on ligand binding affinity and concentration, and is most often incomplete. The high concentration of buffer molecules, crystallization cocktail components, etc. means that these molecules may often outcompete the desired ligand, especially if the affinity of the protein for the ligand in question is not very high. In the unfortunate situation when the binding of a desired ligand leads to conformational changes that are incompatible with the packing of the protein in the crystal lattice, crystallization may be hindered even when the ligand affinity is high. In this case, a less likely complex with common buffer molecules may crystallize instead. As a final hurdle, data collection at cryogenic temperatures requires the addition at high concentration of cryo-protectant molecules that often resemble, and therefore outcompete, the native ligand. Although clearly very important, issues on ligand binding to proteins have only recently come into focus (see, for example, Pozharski *et al.*, 2013; Weichenberger *et al.*, 2013; Adams *et al.*, 2016).

Thus, the binding of MPD in the ligand-binding cavity of D14 is not surprising; the concentration of MPD is high (10%, v/v) and this compound, commonly used in crystallization experiments, may bind in an open cavity poised for binding a ligand—it may even outcompete a ligand initially bound. To avoid MPD binding in the ligand-binding cavity, extensive crystallization screening was carried out with alternative precipitating agents. Well diffracting crystals of apo *Os*D14 were obtained using PEG 6000 as a precipitating agent at concentrations of ~8–20% and in the pH range 6.5–9.5. Because of the slower spontaneous hydrolysis of SL analogue compounds at lower pH, crystallization of ligand complexes of *Os*D14 was generally performed at pH 6.5. A range of compounds were tried: a racemic mixture of GR24 and four other analogues of various sizes (see Supplementary Fig. S1). A mutation of the catalytic Ser147 was introduced and crystallization of the mutant S147A was performed in the same manner. This resulted in a number of diffraction data sets and corresponding structures; a selection is presented in Supplementary Table S1. Analysis of the potential ligand structures showed only minor structural changes: the overall structures were very similar and there were no major conformational transitions [root-mean-square deviations (rmsds) to the MPD-bound structure (PDB id 6elx) 0.15–0.56 Å for 267 Cα atoms of one molecule, Supplementary Table S1]. There was spurious additional density in the ligand-binding cavity indicating the binding of a compound (or a mixture of compounds) at low occupancy. This is shown for structures 5–18 and the mutant S147A (Supplementary Table S1), which were soaked with Compound C and co-crystallized with Compound B, respectively (Supplementary Fig. S1). In 5–18, the extra density extends from Ser147 into the binding cavity and defines a binding site lined by residues Phe78, Phe176, Phe186, Trp205, Tyr209, Cys241, Val244, Phe245, Ser270, and His297 (Supplementary Fig. S2A). The density partly overlaps with the binding site for MPD in the deposited structure (6elx) and we suspect the partial binding of the cryoprotectant glycerol in this structure. However, the contrast to the well-defined density of MDP, which binds at high occupancy, although it is held mainly by solvent-mediated contacts, is striking. As in the MPD-bound structure, Ser270 and Cys241 have double conformations but, in contrast to the MPD-bound structure, the side chain of Ser147 also has two conformations in the C-soaked complex. There is extended extra electron density on the Oγ atom, indicating the binding of a compound to one of the conformers. It is not possible to determine the nature of the bond, covalent or non-covalent, given the low occupancy. As expected, S147A lacks extra density at residue 147, and the density in the cavity appears more continuous (Supplementary Fig. S2B).

Failure to detect distinct binding of SL analogues (or hydrolysis products) to D14 may be explained in several ways. The low occupancy may be explained by low concentration of the SL analogue because of its low solubility in water. Also, if crystal lattice forces do not support the conformational transition required for SL binding, the compound may not be able to bind, or crystallization components may bind in its stead. Finally, if our construct is able to (slowly) hydrolyse the compounds (as already reported for identical constructs, e.g. Zhao *et al.*, 2015), there may have been a mixed binding of hydrolysed and non-hydrolysed compounds in the cavity as well as glycerol and other compounds from the crystallization mixture. We can only speculate on this possibility, because the activity of our construct was not monitored. In spite of this caveat, detailed re-analysis of SL structures deposited in the PDB (see below) shows that these may be common problems, also when structures are obtained from proteins whose activity in solution had been experimentally confirmed before crystallization.

### Comparison with other SL receptor structures

The interaction of MPD with our *Os*D14 is similar to that of MPD in the model of *O. sativa* D14 presented by Kagiyama *et al.* (2013; PDB id 3w04) (Fig. 2B). The two models superimpose with an rmsd of 0.337 Å for 262 Cα atoms (rmsd 0.680 Å for 526 Cα atoms of the two molecules in the asymmetric unit). Electron density for MPD in crystal structure 3w04 is visible in both molecules (A and B) and the binding of the ligand is stabilized by contacts of O4 with the hydroxyl group of Tyr209 and by solvent-mediated hydrogen bonds between O2 of MPD and Oγ of Ser147 and Nε2 of His297.

**Fig. 3.**
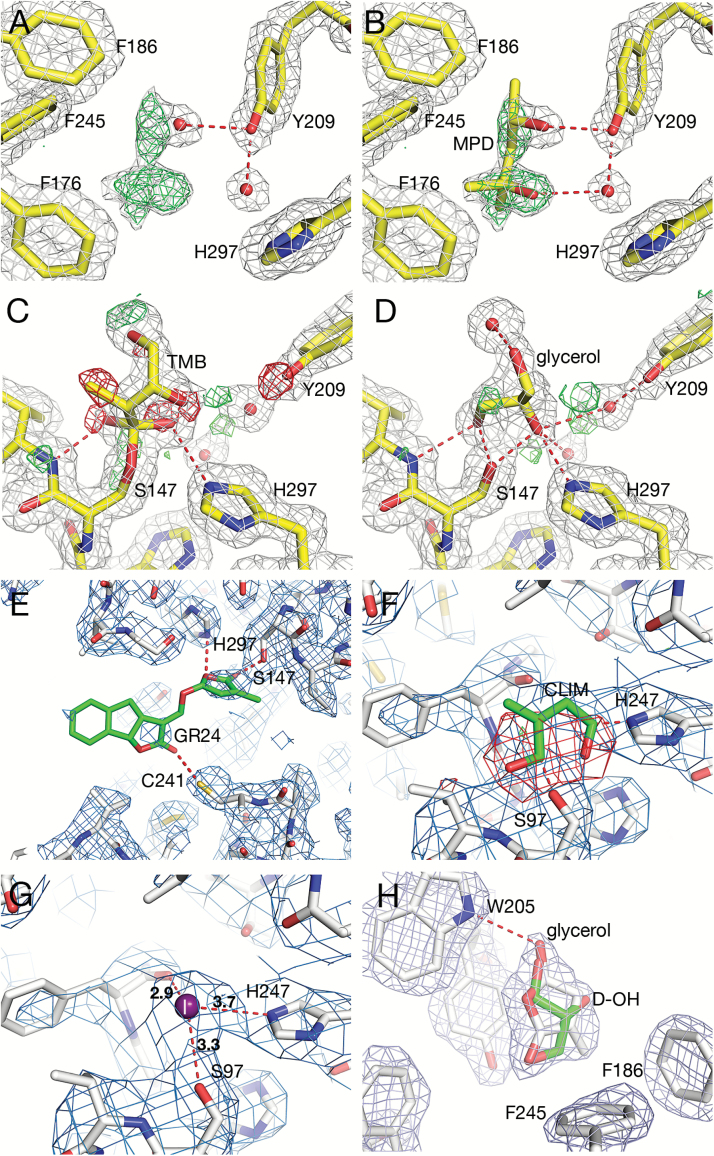
Comparison of ligand binding in diverse D14 structures. Numbering of residues in (A–E) and (H) refers to the full-length *O. sativa* sequence, whereas (F) and (G) show the *A. thaliana* D14 with a shorter sequence. Dotted lines indicate atoms within hydrogen-bonding distance. Bonding distances are in Å. (A) Positive density was detected in the ligand-binding cavity of *O. sativa* D14 (PDB id 4ih9) close to a water molecule. 2*mF*_o_–*DF*_c_ map contoured at 1.0 sigma (white), and *mF*_o_–*DF*_c_ map contoured at 3.0 sigma (green). (B) Same as (A) with MPD built into the vacant density, replacing water. (C) *O. sativa* D14 (PDB id 4iha; Zhao *et al.*, 2013) with its degradation intermediate, 2,4,4,-trihydroxy-3-methyl-3-butenal (TMB). 2*mF*_o_–*DF*_c_ map contoured at 1.0 sigma (white) and *mF*_o_–*DF*_c_ contoured at 3.0 sigma (green) and –3.00 sigma (red) calculated using submitted co-ordinates. Note the red mesh indicating that these modelled atoms are not present in the crystal. (D) Glycerol built into the TMB site. 2*mF*_o_–*DF*_c_ map contoured at 1.0 sigma (white) and 2*mF*_o_–*DF*_c_ contoured at 3.0 sigma (green) and –3.0 sigma (red) calculated after 20 cycles of refinement in Refmac5. This shows a better fit to the electron density than that in (C). (E) *O. sativa* D14 (PDB id 5dj5, Zhao *et al.*, 2015) with GR24 (green) modelled into the ligand-binding cavity. Omit 2*mF*_o_–*DF*_c_ map (blue) calculated after removing the ligand. The map was contoured at 1.0 sigma. Note the weak density for the ligand. (F) *A. thaliana* D14 receptor in a complex with the proteins D3 from *O. sativa* and ASK1 from *A. thaliana* (PDB id 5hzg; Yao *et al.*, 2016). The GR24 hydrolysis intermediate, CLIM (green), modelled into the ligand-binding cavity. 2*mF*_o_–*DF*_c_ map contoured at 1.0 sigma (blue) and *mF*_o_–*DF*_c_ map contoured at –3.0 sigma (red) calculated from submitted co-ordinates. Note the red mesh indicating that these modelled atoms are not present in the crystal. (G) Iodide ion (purple sphere) modelled into the CLIM site. 2*mF*_o_–*DF*_c_ map contoured at 1.0 sigma (blue). This shows a reasonable fit to the electron density, and no peaks were found in the *mF*_o_–*DF*_c_ maps contoured at ±3.0 sigma. (H) *O. sativa* L. cv. Shiokari D14 (PDB id 3wio; Nakamura *et al.*, 2013). 2*mF*_o_–*DF*_c_ map contoured at 1.0 sigma calculated from submitted co-ordinates. Glycerol (green) was modelled into the density at the opening of the cavity and superposed on the D-OH co-ordinates (in white). This shows that glycerol fits equally well as D-OH to the density.

MPD seems poised to bind in the ligand-binding cavity of D14. In the model of the *O. sativa* apo D14 (PDB id 4ih9; Zhao *et al.*, 2013), extensive positive electron density was detected in the *mF*_o_–*DF*_c_ map in one of the monomers (named B) in the asymmetric unit (Fig. 3A). The density, which extends from a solvent molecule (water B463) into the ligand-binding pocket, may be interpreted as MPD [present at 5% (v/v) during crystallization]. MPD modelled (Fig. 3B) at this site (replacing water B463) would be held in place by hydrogen bonds from O4 to the hydroxyl of Tyr209(159) (residue numbers refer to the numbering of the full-length rice sequence, whereas numbers in parentheses refer to the number in the PDB co-ordinate file) and by a solvent-mediated hydrogen bond between MPD O2 and the hydroxyl of Tyr209(159).

In a related structure (PDB id 4iha; Zhao *et al.*, 2013), covalent binding of a putative degradation intermediate, 2,4,4-trihydroxy-3-methyl-3-butenal (TMB), to *O. sativa* D14 was described. However, analysis of electron density maps (2*mF*_o_–*DF*_c_ and *mF*_o_–*DF*_c_) calculated from deposited data indicates a less than satisfactory fit of the intermediate to the electron density. In one subunit (named A), electron density is lacking (negative electron density indicating parts of the model that are not supported by the data) for O2, O4, and C5 of TMB (red mesh in Fig. 3C). In the second subunit (named B), there is negative electron density for O2 and O4 of the intermediate and there is extra density (positive density indicating features present in the data that are not accounted for by the model) close to C4 (not shown). TMB is the hydrate and open form of D-OH and is likely to co-exist with D-OH. Our analysis of the electron density maps indicates that a mixture of TMB and D-OH may fit better in the density. Alternatively, glycerol [present at 20% (v/v) during cryo-cooling] may also bind at the same site. Glycerol (Fig. 3D) would be held in place by an extensive network of hydrogen bonds: between O1 and one water molecule, between O2 and Oγ of Ser147(97), Nε2 of His297(247), and two water molecules, and between O3 and Oγ of Ser147(97) and the backbone N atoms of Val148(98) and Phe78(28). The conclusion is that glycerol is a more likely ligand than TMB in the crystal structure deposited as 4iha, but the presence of some small positive peaks (green) in the difference density map indicates that a mixture of the two ligands may bind at the site.

Glycerol was also modelled in the internal cavity of petunia DAD2 (PDB id 4dnp) by Hamiaux *et al.* (2012). Inspection of electron density maps calculated from the deposited model reveals that glycerol is only partially supported by electron density; O1 is supported by electron density, but lacks interaction with the protein, whereas O2 is supported by electron density and hydrogen-bonds to Nε2 of the catalytic His246 (not shown). There is no density for the O3 hydroxyl, which in the model would be hydrogen-bonded to the catalytic Ser96 and to two solvent molecules, but apparently the placing of the O3 hydroxyl was inferred from another crystal structure (see the supplementary experimental procedures in Hamiaux *et al.*, 2012).

Binding of the SL analogue GR24 to rice D14 was detected in solution by Zhao *et al.* (2015). To capture the complex in the crystal, GR24 was added at a high molar ratio to a dilute solution of D14 that was subsequently concentrated for crystallization. Electron density maps calculated from the structure show only very weak density in the ligand-binding cavity (PDB id 5dj5) and there are no significant conformational differences between the crystal structures with or without GR24, as would be expected if a ligand was bound. The electron density map calculated after removing GR24 from the structure shows some faint electron density close to the hydroxyl group of the catalytic Ser147(97), whereas there is no density indicating the presence of the ABC moiety (Fig. 3E). The very weak residual density in the omit maps indicates only partial occupancy of a (small) potential ligand, which in this case would be impossible to identify. Because of the low solubility of GR24 in water, it may fall out of solution during concentration/crystallization. It may also be that the crystal packing does not support the conformational changes required to bind GR24 and that therefore apo D14 was crystallized, without the ligand being able to bind.

A conformational transition was detected by Yao *et al.* (2016) in the crystal structure (PDB id 5hzg) of a part of the SCF degradation complex involved in SL signal propagation, where D14 is shown to undergo a shape change that closes its large open ligand-binding cavity. The crystallized complex consists of the proteins *A. thaliana* D14, *O. sativa* D3, and *A. thaliana* SCF-component ASK1 (Arabidopsis Skp1-like). The conformational transition consists of rotational movements, coiling, unfolding, and extending of the four α-helices of the helical cap, resulting in the shrinking of the ligand-binding cavity and the formation of a lid that closes the cavity. This finding contrasts with the results from a crystal structure of a TIR–ASK1 complex active in auxin perception, where auxin does not induce conformational changes, but instead acts as a molecular glue in mediating ligand–receptor interactions (Tan *et al.*, 2007).

A small ligand appears to be trapped in the cavity of D14 and was identified as a GR24 hydrolysis intermediate, named CLIM (covalently linked intermediate molecule), covalently linked to His247, Ser97, or both (Yao *et al.*, 2016). Analysis of electron density maps calculated from co-ordinates of the complex (Fig. 3F) shows that the size of the density peak around the intermediate is too small to accommodate CLIM, leaving a major part (atoms C4, C5, O1, and O2) of the putative intermediate outside of the electron density (blue mesh in Fig. 3F). In addition, a large negative peak in the *mF*_o_–*DF*_c_ difference map (red mesh in Fig. 3F) indicates the presence of atoms in the model which are not present in the crystal. The crystallization solution contained 200 mM KI and 10 mM Pr(III) acetate. We conclude that the most likely ligand is an iodide ion. Insertion of iodide into density followed by 10 cycles of restrained refinement (according to the protocol described by the authors) shows this to be consistent with the shape and position of the density peak (Fig. 3G). However, the modelling of a small compound at the relatively low resolution of the data (3.3 Å) is not straightforward. In addition, refinement appears to be underdetermined; that is, the number of parameters to be refined (62 704) exceeds the number of unique reflections (49 694). Refinement of grouped *B*-factors (one per residue instead of one per atom) would be recommended in this case (see, for example, p. 20 of Wlodawer *et al.*, 2008). Acetate was present at 10 mM concentration in the crystallization solution; therefore, acetylation of the (reactive) catalytic serine may also be considered. If CLIM was not bound in the active site cavity, then what triggered the conformational transition? One possibility is that GR24 may be initially bound and fragmented, thereby triggering the shape change that allows the ternary complex D14–D3–ASK1 to form. The intermediate fragment may subsequently be replaced by iodide ion, which was present at high concentration in the crystallization cocktail.

From all of the above, the fate of an SL or an SL analogue binding in the cavity of the receptor is still to be considered unknown. What is the exact structure of the putative intermediate and how does it interact with the receptor? Based on a structure of rice D14 (PDB id 3wio; Nakamura *et al.*, 2013) a mechanism was suggested where D-OH binds not at the bottom of the ligand-binding cavity near the catalytic triad but at the opening of the cavity. D-OH is suggested to act as a plug for the catalytic cavity and to induce a hydrophobic patch to facilitate the interaction of D14 and its target proteins. Electron density maps calculated from the submitted co-ordinates show a reasonable fit to the electron density of a compound, H3M, that mimics D-OH but lacks the required planarity of the five-membered ring (Fig. 3H). H3M was modelled in one of the molecules in 3wio, whereas the ligand-binding cavity is empty in both molecules A and B in the apo structure (PDB id 3vxk). There is no conformational change detected in comparing the structures of the apo enzyme and the H3M ligand complex. However, modelling of glycerol [present at 10% (v/v) during purification] shows that this compound also fits the electron density at the opening of the cavity (Fig. 3H). There is also the possibility that a mixture of compounds may bind. Thus, also in this case, the nature of the bound compound may be considered unknown.

### Conclusions

To identify the active form(s) of SL in plants and their molecular mode of action has long been a major technical challenge and a hotspot in SL biology. However, the unusual nature of the SL hormone receptor, simultaneously able to perceive and hydrolyse (destroy) the hormone, has made this a formidable task. There is evidence from studies in solution suggesting that the receptor may cleave the SL and form a covalent bond to the hormone fragment (de Saint Germain *et al.*, 2016; Yao *et al.*, 2016). This in turn may trigger a dramatic change in shape of the receptor, thereby forming surfaces that can interact with signalling partners. The rate of cleavage appears to be extremely slow, and may in fact stall at the first turnover (Hamiaux *et al.*, 2012; de Saint Germain *et al.*, 2016). The chemical nature of the hormone fragment, supposedly a small molecule, ~5 carbon atoms in size, is another obstacle in the way of determining a structure of a complex between the receptor and its hormone. Results from our attempts at capturing a complex of D14 with SL analogues and our analysis of available structures of SL receptor complexes indicate that in most cases the modelled compound may either be outcompeted by common buffer and crystallization compounds, or may bind at very low occupancy, making modelling extremely uncertain. It is also possible that the conformational transitions accompanying SL binding may be incompatible with the packing of the protein in the crystal lattice, leading to the apo protein being titrated out of solution (meaning that the subpopulation of the apo enzyme was preferentially crystallized, leaving behind any complex that incorporated the ligand). We conclude that the active forms of SL and their interaction with the SL receptor are yet to be identified.

## Supplementary data

Supplementary data are available at *JXB* online.

Table S1. Data collection and refinement statistics for D14–ligand complex searches.

Fig. S1. Strigolactone analogues used in crystallization experiments.

Fig. S2. Electron densities in the ligand-binding cavity of *Os*D14 native and S147A mutant proteins.

Supplementary Material

## Abbreviations:

CLIM: covalently linked intermediate molecule
D14: DWARF14
GID1: Gibberellin Insensitive Dwarf1
MAX: MORE AXILLARY GROWTH
MPD: 2-methylpentanediol
rmsd: root-mean-square deviation
SCF: Skp, Cullin, F-box containing complex
SL: strigolactone; SMAX1, SUPPRESSOR OF MAX2 1
SMXL: SMAX1-LIKE
TMB: 2,4,4-trihydroxy-3-methyl-3-butenal.
