# Supplementary material for: The elusive ligand complexes of the DWARF14 strigolactone receptor
Source: J Exp Bot. 2018 Jan 31;69(9):2345–54. doi: 10.1093/jxb/ery036 (PMC5913616; doi:10.1093/jxb/ery036)
Supplement: Supplementary Material [file ery036_suppl_supplementary_material.pdf]

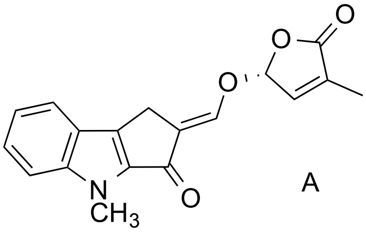

A

(*R*,*E*)-4-methyl-2-(((4-methyl-5-oxo-2,5-dihydrofuran-2-yl)oxy)methylene)-1,2-dihydrocyclopenta[*b*]indol-3(4*H*)-one

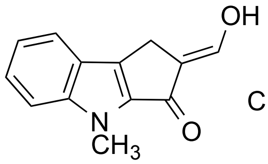

C

(*E*)-2-(hydroxymethylene)-4-methyl-1,2-dihydrocyclopenta[*b*]indol-3(4*H*)-one

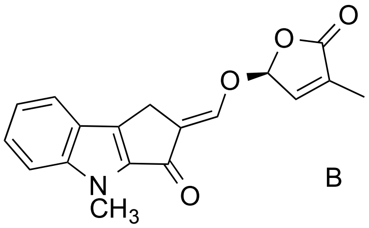

B

(*S*,*E*)-4-methyl-2-(((4-methyl-5-oxo-2,5-dihydrofuran-2-yl)oxy)methylene)-1,2-dihydrocyclopenta[*b*]indol-3(4*H*)-one

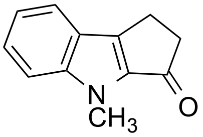

D

4-methyl-1,2-dihydrocyclopenta[*b*]indol-3(4*H*)-one

Supplementary Figure S1. Strigolactone analogues used in crystallisation experiments.

Supplementary Figure S2. Ligand binding cavity of *Os*D14, showing 2*mFo* - *DFc* map contoured at 1 σ (white), and *mFo* - *DFc* map contoured at 3 σ (green) in (**A**) crystal D14 5-18, and (B) crystal D14 S147A.

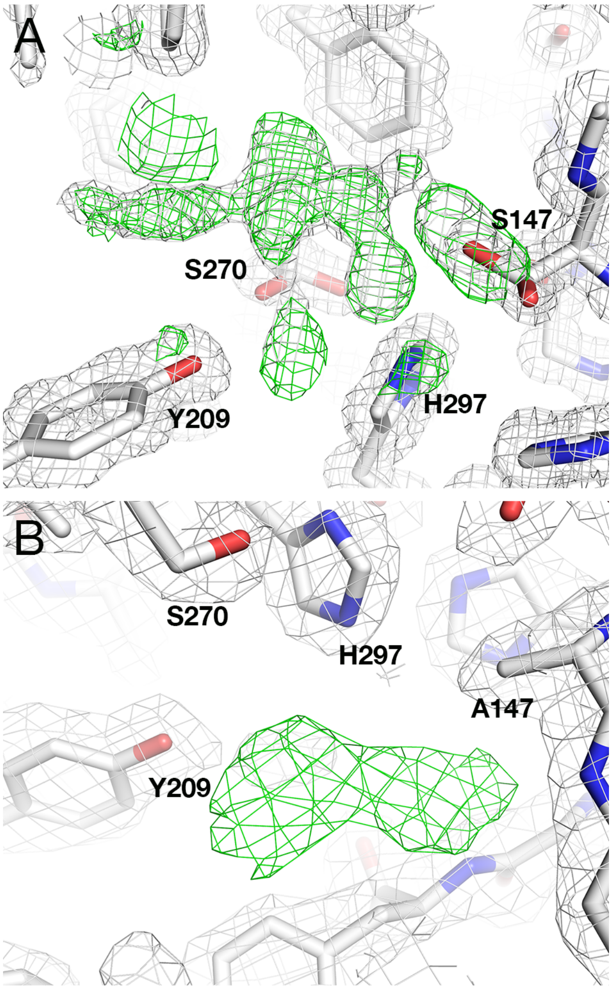

Supplementary Table S1. Data collection and refinement statistics for D14 ligand complex searches. Statistics for the highest-resolution shell are shown in parentheses.

Crystal id.	D14 1-3<br>GR24 soaked	D14 3-11<br>B soaked	D14 4-2<br>A co-cryst.	D14 5-18<br>C soaked	D14 S147A<br>C soaked
Crystallisation conditions	0.1 M Hepes pH 7.0, 10% PEG 6000	0.1 M Hepes pH 7.0, 20% PEG 6000	0.1 M Bis-Tris propane pH 6.5, 20% PEG 6000	0.1 M Bis-Tris propane pH 6.5, 8% PEG 6000	0.1 M Bis-Tris propane pH 6.5, 8% PEG 6000
**Data collection**					
R.m.s.d. to 6elx (Å)	0.124	0.258	0.325	0.145	0.561
Wavelength	0.93222	0.97247	0.87300	0.97625	0.87260
Resolution range	44.33 -1.5 (1.554 -1.5)	31.39 - 1.5 (1.554 - 1.5)	38.79 - 1.6 (1.657 - 1.6)	44.74 - 1.151 (1.192 - 1.151)	37.82 - 2.15 (2.227 - 2.15)
Space group	$P2_12_12_1$	$P2_12_12_1$	$P2_12_12_1$	$P2_12_12_1$	$C2_1$
Unit cell	48.03 88.66 118<br>90.0 90.0 90.0	47.91 88.8<br>118.13<br>90.0 90.0 90.0	46.34 70.97<br>70.76<br>90 91.331 90.0	48.31 88.71<br>118.55<br>90.0 90.0 90.0	77.88 49.62<br>69.19<br>90.0 106.2 90.0
Unique reflections	80204 (7162)	80438 (7525)	59047 (5019)	179744 (17385)	13976 (1398)
Completeness (%)	98.5 (88.6)	98.7 (93.6)	97.6 (83.0)	100.0 (96.0)	99.75 (99.15)
$R_{meas}{}^{a}$	0.08148 (0.998)	0.05603 (1.408)	0.1153 (0.9698	0.1575 (0.9687)	0.2062 (1.155)
Wilson B	15.1	20.1	13.4	13.4	32.5
**Refinement**					
No reflections in refinement	80204 (7162)	80430 (7525)	59033 (5012)	159292 (15670)	13960 (1395)
No reflections R_{free}	4012 (359)	4027 (379)	2002 (168)	7948(769)	698 (70)
$R_{work}{}^{b}$	0.1792 (0.2835)	0.1881 (0.3653)	0.1776 (0.2977)	0.1981 (0.2865)	0.1826 (0.3121)
$R_{free}{}^{c}$	0.2052 (0.2988)	0.2218 (0.3898)	0.2105 (0.3512)	0.2137 (0.2948)	0.2500 (0.4056)
No. of atoms	4830	4868	4675	4988	2299
Proteins	4217	4221	4259	4199	2089
Ligands	48	21	18	16	54
Waters	317	626	398	719	156
Protein residues	535	535	535	534	268
R.m.s.d. from ideal					
Bond lengths (Å)	0.006	0.006	0.006	0.005	0.008
Bond angles (Å)	1.12	1.08	0.79	1.14	1.23
Ramachandran analysis[d]					
..Favoured (%)	98.0	98.0	98.7	98.0	97.0
Allowed (%)	1.6	2.0	1.3	2.2	3.0
Outliers (%)	0.36	0.36	0.0	0.18	0.0
Rotamer outliers (%)	0.87	1.3	0.65	1.3	3.1
Clashscore	3.14	2.92	1.04	3.90	7.86
Average B-factor	20.77	27.74	16.39	19.22	25.60
protein	19.20	26.15	15.51	17.31	24.97
ligands	38.50	49.52	30.61	34.77	35.10
solvent	31.22	37.74	25.22	29.32	30.79

[a]$R_{meas} = \sum_h \sum_l (n_h/n_h\text{-}1)^{1/2} \mid I_{hl} - \langle I_h \rangle \mid / \sum_h \sum_l \langle I_h \rangle$ (Evans, 2006; Evans and Murshudov, 2013).
[b]$R_{work} = \sum_{hkl} \mid |F_o| - |F_c| \mid / \sum_{hkl} |F_o|$ where F_o and F_c are the observed and calculated structure factor amplitudes, respectively.
[c]R_{free} calculated from a randomly chosen 5% of all unique reflections.
[d]From *MolProbity* (Chen *et al.*, 2010).
